# Toxicity of Nab-Paclitaxel Compared to Paclitaxel in a Tertiary Hospital in Jeddah, Saudi Arabia: A Retrospective Cohort Study

**DOI:** 10.7759/cureus.39872

**Published:** 2023-06-02

**Authors:** Suha A Alhebshi, Safaa M Alsanosi, Hamsa S AlQashqri, Yosra Z Alhindi, Ghazi A Bamagous, Nahla A Ayoub, Alaa H Falemban

**Affiliations:** 1 Pharmaceutical Services, King Abdulaziz University Hospital, Jeddah, SAU; 2 Pharmacology and Toxicology, Faculty of Medicine, Umm Al-Qura University, Makkah, SAU; 3 Community and Family Medicine, Faculty of Medicine, Umm Al-Qura University, Makkah, SAU

**Keywords:** albumin-bound, nanoparticle, pancreatic cancer, breast cancer, nab-paclitaxel, paclitaxel, toxicity

## Abstract

Background

Nanoparticle albumin-bound paclitaxel has been developed to avoid the toxicities associated with Cremophor-solved paclitaxel. Although many studies confirm this hypothesis, there is recent evidence showing no difference between paclitaxel and nab-paclitaxel in their efficacy and safety profile. This study further assesses the toxicity of both paclitaxel and nab-paclitaxel in adult patients with breast and pancreatic cancer in a tertiary hospital in Jeddah, Saudi Arabia. These toxicities include neutropenia, anaemia, and effects on kidney and liver functions.

Methods

The study is a retrospective cohort study done at King Abdulaziz University Hospital, Jeddah, Saudi Arabia, from January 2018 to December 2021, conducted on patients diagnosed with breast or pancreatic cancer treated with paclitaxel or nab-paclitaxel.

Results

There is a statistically significant difference between the two groups in developing anaemia, renal, and liver toxicity (P<0.05). On the other hand, there are no statistically significant differences in developing neutropenia between the two groups (P=0.084).

Conclusions

Nab-paclitaxel might not be better than paclitaxel in reducing the risk of neutropenia, anaemia, and liver toxicity, as predicted. Nevertheless, both medications require that the patient's renal functions be monitored during the treatment. Further studies conducted in multiple oncology centres with a larger sample are needed to evaluate the toxicity of paclitaxel and nab-paclitaxel in adult patients with breast and pancreatic cancer.

## Introduction

Cancer is a disease characterised by uncontrollable cell proliferation and spread. There were an estimated 1.8 million diagnoses and more than 600,000 deaths from cancer in the USA in 2020 [1]. Breast cancer is the leading type of cancer in Saudi Arabia [2]. According to the most recent survey of cancer-related mortality, it is the 10th-highest cause of death among Saudi women [3]. Pancreatic cancer was the 10th most common malignancy among Gulf Cooperation Council (GCC) nationals aged 60 to 74 years old, whereas in Saudi Arabia, the rates of pancreatic cancer were significantly higher among males than females [4].

Due to their ability to inhibit the cell proliferation cycle, taxanes are the most utilised chemotherapeutic medicines to treat various cancer types [5]. The first approved medicine of this type, paclitaxel, is now the established therapy for early-stage metastatic breast cancer [6]. Due to its limited solubility in water, it is formulated in polyoxyethylated castor oil (Cremophor) or dehydrated ethanol. However, this form of paclitaxel has been reported to cause a wide range of side effects, mainly bone marrow suppression, allergic responses, and neurotoxicity [7-9].

Because of this, paclitaxel has been modified utilizing nanotechnology to become nanoparticle albumin-bound paclitaxel (nab-paclitaxel) [10]. This solvent-free nanometer-sized form of paclitaxel was first developed in 1992 to avoid the toxicities associated with Cremophor. It can be administered with a shorter infusion schedule and no pre-medications, and it has been used to treat breast cancer by The Food and Drug Administration (FDA) since 2005 [5,11].

Indeed, some clinical trials and meta-analyses showed the superiority of nab-paclitaxel to solvent-based paclitaxel, in terms of efficacy and safety, in patients with metastatic breast cancer and patients with pancreatic cancer [12-14]. However, other studies have been done and show that there is no difference between paclitaxel and nab-paclitaxel in their efficacy and safety profile.

In one meta-analysis, side events such as neutropenia were found to be equivalent across the paclitaxel and nab-paclitaxel groups (P=0.26) [13]. Alternatively, according to another study, the incidences of peripheral neuropathy and neutropenia were higher in the nab-paclitaxel-gemcitabine group (38% in the nab-paclitaxel-gemcitabine group vs. 27% in the gemcitabine group) [12]. In a multi-centre, open-label study, patients with breast cancer had 30.6% neutropenia of grade 3 or higher in the nab-paclitaxel arm and 19.7% in the paclitaxel arm [15]. In a meta-analysis, nab-paclitaxel was associated with more frequent sensory neuropathy than paclitaxel and docetaxel in treating metastatic breast cancer [16]. 

This study aims to assess the toxicity of paclitaxel and nab-paclitaxel in adult patients with breast and pancreatic cancer in a tertiary hospital in Jeddah, Saudi Arabia. These toxicities include neutropenia, anemia, and effects on liver functions. In addition, one of our primary goals is to find a relation between nephrotoxicity and paclitaxel or nab-paclitaxel since we have not found studies assessing the relation between nephrotoxicity and paclitaxel or nab-paclitaxel.

## Materials and methods

This study is a comparative retrospective cohort study conducted at King Abdulaziz University Hospital, Jeddah, Kingdom of Saudi Arabia, approved by the Ethics Committee of the same hospital (approval number HA-02-J-008).

Patients

All male and female patients older than 18 years who contacted the day care unit at King Abdulaziz University Hospital in the period between January 2018 to December 2020 were included. Patients were included if they were diagnosed with breast or pancreatic cancer and treated with paclitaxel or nab-paclitaxel. Patients could also have received fluorouracil or gemcitabine as adjuvant therapy.

Exclusion criteria were patients < 18 years, patients diagnosed with cancer different from breast or pancreatic even if they were treated with paclitaxel or nab-paclitaxel, and patients diagnosed with breast or pancreatic cancer not treated with paclitaxel or nab-paclitaxel.

Study endpoint

The primary objective was to compare the toxicity of paclitaxel and nab-paclitaxel in adult patients with breast and pancreatic cancer. These toxicities include neutropenia, anaemia, nephrotoxicity, and liver toxicity.

Statistical analysis

Data were transferred to IBM SPSS Statistics (IBM Corp., Armonk, NY, USA) for analysis. Unpaired T-tests, chi-square, and descriptive analysis were used to analyse the relationship between different variables in each group. Unpaired T-tests were used to analyse the relation between neutropenia, haemoglobin level, creatinine clearance, and aspartate aminotransferase (AST) and alanine aminotransferase (ALT) in each group (paclitaxel and nab-paclitaxel groups). Chi-square determined the P-value between the two groups; P<0.05 indicates statistically significant differences. Descriptive analysis was used to determine the mean, median, and average.

## Results

Patients

One hundred twenty-one adult patients (24 male and 97 female) >18 years old, aged 35 to 60 years old, were treated at King Abdulaziz University Hospital in Jeddah from January 2018 to December 2021. These patients were diagnosed with breast cancer (n=84) or pancreatic cancer (n=37) regardless of clinical stages (Table 1).

**Table 1:** Demographic data of study population

Total Number of patients		121	
Age		55+13.6	
Gender		Female	Male
		97	24
Breast cancer			
	No. Patients	84	
	Paclitaxel	70	0
	Nab –paclitaxel	14	0
Pancreatic cancer			
	No. Patients	37	
	Paclitaxel	13	2
	Nab –paclitaxel	0	22

Eighty-four patients had been treated with paclitaxel. There were 70 patients with breast cancer (all female) and 15 with pancreatic cancer (two males and 13 females). Thirty-seven of these patients (14 of whom had breast cancer and 22 of whom had pancreatic cancer) had been treated with nab-paclitaxel.

Safety and toxicity

**Figure 1:**
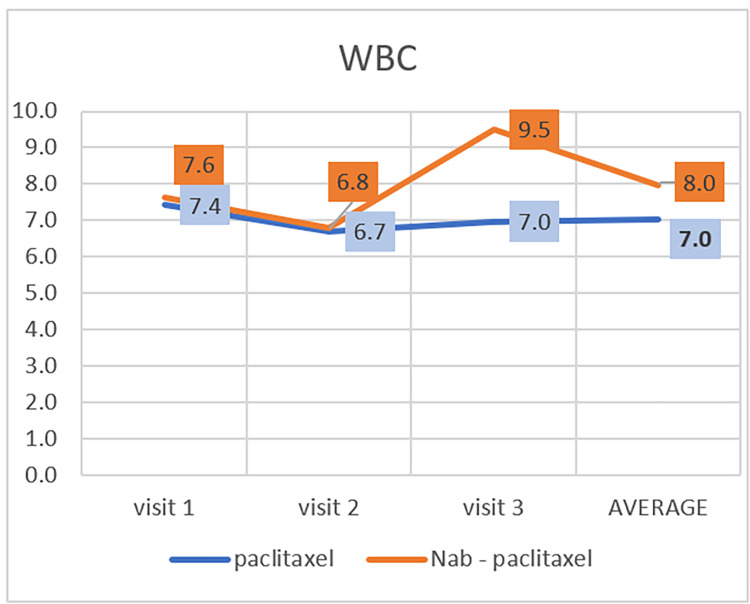
Effect of paclitaxel and nab-paclitaxel on white blood cells (WBC)

The normal range of white blood cells (WBC) in humans is 4.5-11.5 K/µL [17]. In the paclitaxel group, the average WBC in patients was 7.4 in the first visit; it decreased to 6.6 in the second and slightly increased to 6.9 in the third visit. In the nab-paclitaxel group, the average WBC in patients was 7.6 in the first visit; it decreased to 6.7 in the second visit and increased to 9.5 in the third visit (Table 2, Figure 1). There is no statistically significant difference in developing neutropenia between these two groups (P=0.084). Also, there is no clinically significant difference in developing neutropenia between these two groups because the WBC in both groups is within the normal range.

**Table 2:** Analyses of blood collected during three visits for patients WBC: white blood cells, ALT: alanine aminotransferase, AST: aspartate aminotransferase

	Visit 1			Visit 2			Visit 3			P-value
	Mean ± Standard deviation									
WBC										
Paclitaxel Group	7.42	±	3.46	6.69	±	2.56	6.98	±	3.99	0.084
Nab-paclitaxel Group	7.63	±	2.75	6.79	±	2.30	9.50	±	4.92	
Haemoglobin										
Paclitaxel Group	11.04	±	1.39	10.91	±	1.28	10.46	±	1.52	0.001
Nab-paclitaxel Group	11.65	±	2.69	11.23	±	1.81	11.15	±	2.23	
Creatinine Clearance										
Paclitaxel Group	44.00	±	15.30	44.06	±	14.29	44.52	±	15.29	0.030
Nab-paclitaxel Group	38.23	±	12.17	38.00	±	12.96	37.89	±	13.70	
ALT										
Paclitaxel Group	32.87	±	25.73	37.18	±	34.49	35.74	±	30.41	0.008
Nab-paclitaxel Group	29.28	±	18.04	32.67	±	14.01	30.61	±	15.49	
AST										
Paclitaxel Group	30.49	±	31.57	30.54	±	31.63	30.04	±	29.90	0.005
Nab-paclitaxel Group	28.78	±	17.08	34.61	±	34.08	38.28	±	30.84	

**Figure 2:**
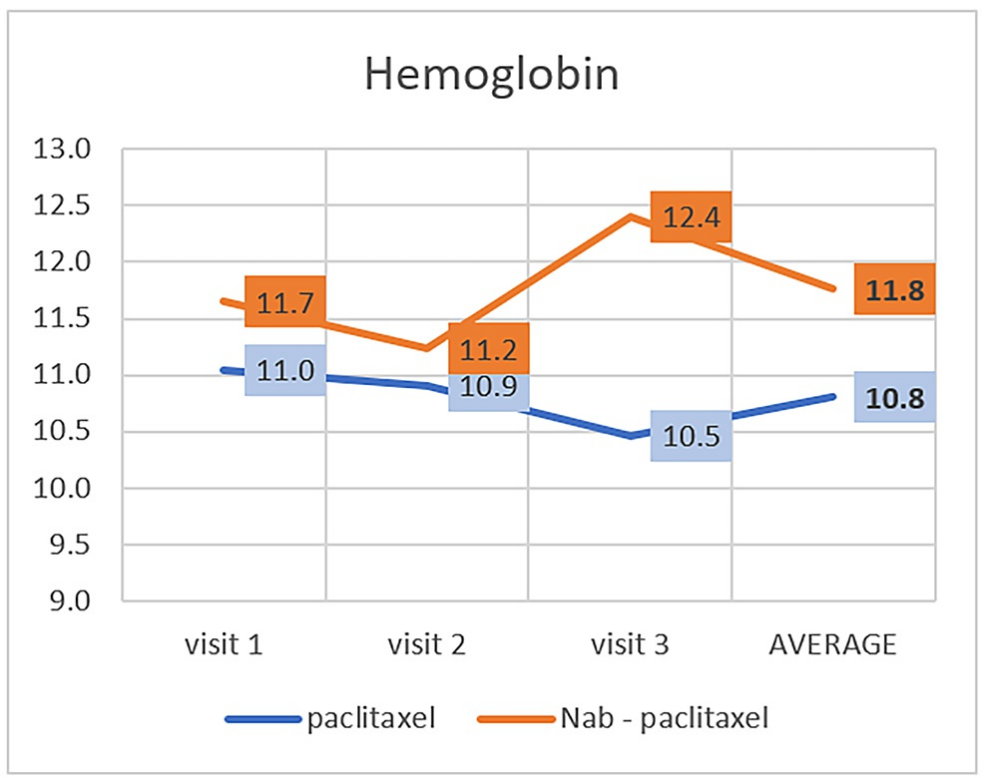
Effect of paclitaxel and nab-paclitaxel on haemoglobin

The expected haemoglobin level in males is 14 -16 g/dL, and in females, 12-16 g/dL [18]. In the paclitaxel group, the average haemoglobin level was 11 in the first visit; it decreased in the second and third visits. In the nab-paclitaxel group, the average haemoglobin level was 11.7 on the first visit. It decreased in the second visit and increased in the third visit (Table 2, Figure 2). There is a statistically significant difference in developing anemia between these two groups (P=0.001).

**Figure 3:**
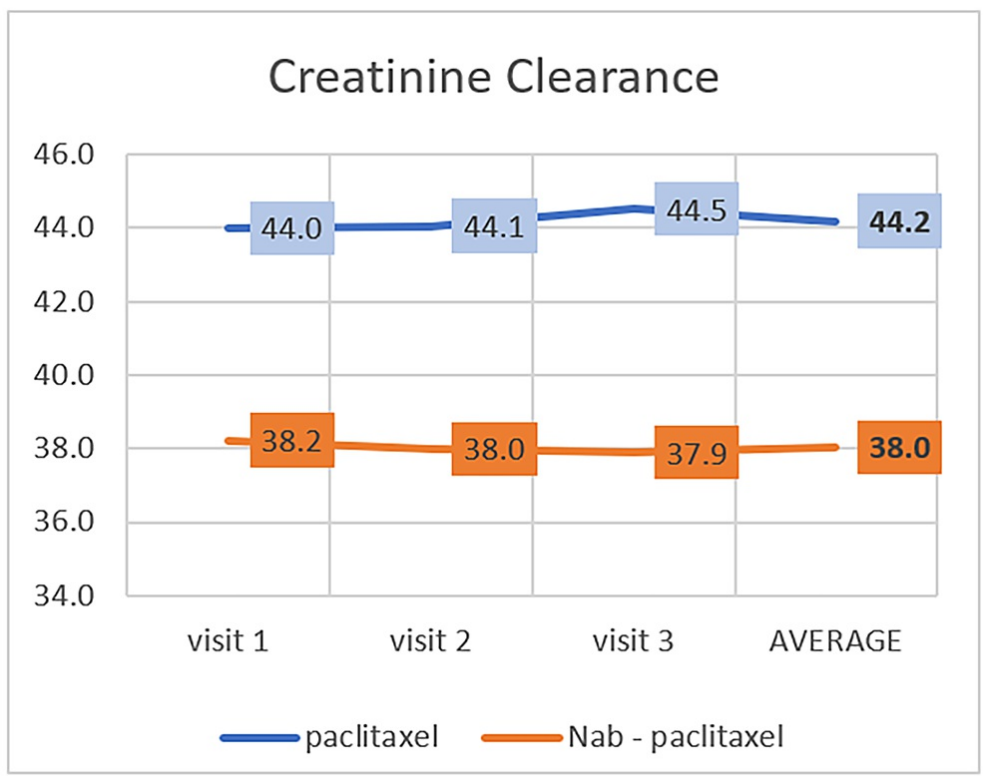
Effect of paclitaxel and nab-paclitaxel on creatinine

The usual range of creatinine clearance is 110 to 150 mL/min in males and 100 to 130 mL/min in females [19]. The average creatinine clearance slightly increased after the first visit in the paclitaxel group. In contrast, in the nab-paclitaxel group, the average creatinine clearance slightly decreased after the first visit (Table 2, Figure 3). There is a statistically significant difference in developing nephrotoxicity after treatment between these two groups (P=0.030).

**Figure 4:**
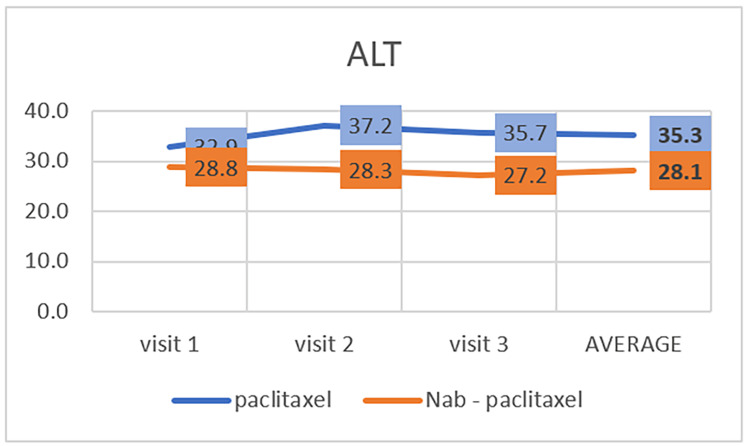
Effect of paclitaxel and nab-paclitaxel on alanine aminotransferase (ALT)

Toxicity of the liver can be assessed by ALT and AST levels; the normal range of ALT is 12-78 IU/L. The average ALT level slightly increased and then decreased in the paclitaxel group. At the same time, in the nab-paclitaxel group, the average ALT level decreased throughout the three visits (Table 2, Figure 4). There is a statistically significant difference between these two groups (P=0.008).

**Figure 5:**
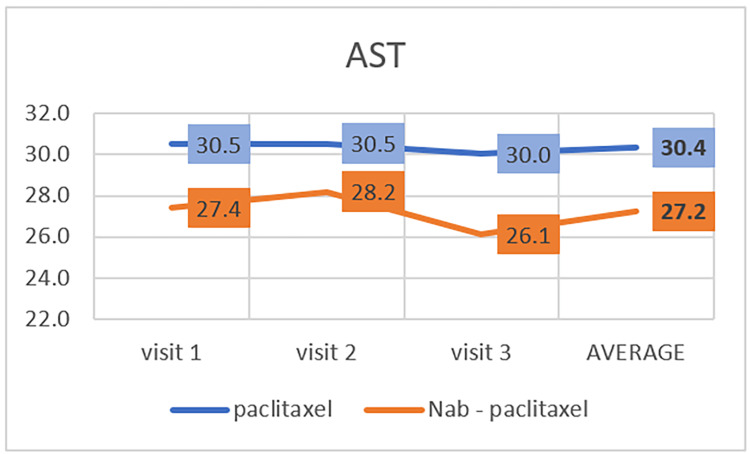
Effect of paclitaxel and nab-paclitaxel on aspartate aminotransferase (AST)

Further, the normal range of AST is 15-37 IU/L. The average AST level decreased during the third visit in the paclitaxel group. In contrast, in the nab-paclitaxel group, the average AST level decreased throughout the three visits (Table 2, Figure 5). There is a statistically significant difference between these two groups (P<0.05).

## Discussion

Paclitaxel and nab-paclitaxel have been approved by the FDA for treating different types of cancers, mainly breast cancer, the second most common cancer in Saudi women, and pancreatic cancer, the fourth highest cancer death cause in the US [20]. Breast cancer occurs less in men than in women [21], which might explain why the number of males diagnosed with breast cancer was zero in our study.

Neutropenia is one of the side effects associated with nab-paclitaxel. Braiteh et al. reported a 28% incidence in patients with metastatic pancreatic cancer and a 4% incidence in patients with a previous history of chemotherapy [22]. However, in comparison with paclitaxel, neutropenia was equivalent across the paclitaxel and nab-paclitaxel groups, and there was no statistically significant difference between them (P=0.26) [23]. This is similar to our result in this study, as we found no statistical difference between the two groups (P=0.084). In the current study, we noted that the number of WBCs of both groups is in the normal range (4.5-11.5 K/µL). This might be attributed to using a granulocyte colony-stimulating factor (G-CSF) which stimulates the production of WBCs in bone marrow [24]. G-CSFs have been found to reduce the incidence and duration of neutropenia and febrile neutropenia in patients receiving chemotherapy.

Paclitaxel produced anemia in 36%-51% of previously treated patients with metastatic breast cancer [25]. Nab-paclitaxel had the most favourable safety profile, with the lowest incidence of anemia [26]. According to our study, there was a statistically significant difference in the incidence of anemia between the two groups, similar to previous studies; nab-paclitaxel had a lower incidence of anemia (P=0.001). We noticed that the average hemoglobin levels in paclitaxel and nab-paclitaxel groups were between 11-12 g/dL, classified as mild anemia. Most of the patients had such acceptable hemoglobin levels and did not exceed this severity of grade, probably because they were receiving epoetin alfa during their treatment course. Epoetin alfa is erythropoietin, a hematologic growth factor that regulates red blood cells' proliferation, maturation, and differentiation [27].

To the best of our knowledge, no studies assess the relation between renal toxicity and paclitaxel or nab-paclitaxel. As a result of our study, we discovered that an average of the patients in both groups had their creatinine clearance decrease to levels classified as stage 3B, a moderate degree according to the classification of chronic kidney disease [28]. Furthermore, the creatinine clearance levels of patients on nab-paclitaxel were significantly lower than those observed in the paclitaxel group, with a median creatinine clearance of 38.0 and 44.2 mL/minute, respectively (P=0.030). It is unknown whether the finding should be attributed to paclitaxel or other factors such as pre-medication or the indication itself. In a retrospective study, a combination of paclitaxel-cisplatin was attributed to a decrease of more than 25% in creatinine clearance from baseline in 81% of patients with gynecological cancer in contrast to only 29% of patients on cisplatin alone [29]. Further research is required, especially when it comes to long-term outcomes, to determine if paclitaxel and nap-paclitaxel affect renal function in such a way.

Few studies have discussed the relationship between paclitaxel and nab-paclitaxel in hepatic toxicity. One study found a relationship between paclitaxel and hepatic toxicity in patients with breast cancer and showed that paclitaxel could cause an increase in abnormal levels of liver enzymes [30]. In our study, we found higher levels of ALT and AST in patients who took paclitaxel than in those who took the nab-paclitaxel, although the increases were in the normal range. Again, further research is needed to determine whether paclitaxel or nab-paclitaxel treatment affects hepatic toxicity.

Although this study has a small sample size collected from a single oncology centre, it has been conducted in one of the big tertiary hospitals in the western region of Saudi Arabia. In our study, furthermore, nephrotoxicity has been assessed in relation to paclitaxel and nab-paclitaxel. Further studies with a larger sample size conducted in multiple oncology centres are needed to evaluate the toxicity of paclitaxel and nab-paclitaxel in adult patients with breast and pancreatic cancer.

## Conclusions

Nab-paclitaxel (nanoparticle albumin-bound paclitaxel) was first developed to avoid the toxicities associated with Cremophor-solved paclitaxel. However, our study shows that nab-paclitaxel might not be better than paclitaxel in reducing the risk of neutropenia, anaemia, and liver toxicity, as predicted. Nevertheless, both medications require that the patient's renal functions be monitored during treatment. Further comprehensive studies of the toxicity of paclitaxel and nab-paclitaxel in adult patients with breast and pancreatic cancer are required.
